# Ex Vivo Metrics™, a preclinical tool in new drug development

**DOI:** 10.1186/1479-5876-6-5

**Published:** 2008-01-23

**Authors:** C Gerald Curtis, Kevin Bilyard, Hugo Stephenson

**Affiliations:** 1 Bowman Research, Inc., 2570 East Devon Ave, Des Plaines, USA; 2 Nine-TZ Healthcare Ventures, The Post House, Congleton, Cheshire, UK; 3 Quintiles Inc., 5927 S Miami Blvd, Morrisville, USA

## Abstract

Among the challenges facing translational medicine today is the need for greater productivity and safety during the drug development process. To meet this need, practitioners of translational medicine are developing new technologies that can facilitate decision making during the early stages of drug discovery and clinical development. Ex Vivo Metrics™ is an emerging technology that addresses this need by using intact human organs ethically donated for research. After hypothermic storage, the organs are reanimated by blood perfusion, providing physiologically and biochemically stable preparations. In terms of emulating human exposure to drugs, Ex Vivo Metrics is the closest biological system available for clinical trials. Early application of this tool for evaluating drug targeting, efficacy, and toxicity could result in better selection among promising drug candidates, greater drug productivity, and increased safety.

## Introduction

Recently, much attention has been given to productivity and safety issues hampering the development of effective new treatments by the pharmaceutical industry. Drug development is prohibitively expensive, mainly due to high attrition rates during clinical trials [1,2]. In response to this, as well as to recent, high-profile safety and toxicity issues [1,3], many companies have chosen to avoid risk by making incremental improvements to existing products rather than focusing on innovation and expansion based on exploratory research.

Practitioners of translational medicine are addressing the productivity and safety obstacles to drug development by encouraging multidisciplinary debate to surface the right questions and then applying the right tools to derive answers [4]. The right questions are those that direct the next step of drug development by providing sufficient information to support either continuation or termination of the development of a particular drug candidate. The right tools are those that generate reliable, interpretable data to enhance the success rate and productivity of drug development.

Particularly important among tools of translational research are those capable of improving early decision making, such as by determining human relevancy and predictability or by selecting the best drug from among several promising candidates. Although many technologies and approaches, such as attrition rate modeling, proof-of-concept strategies, combinatorial chemistry, and pharmacogenomics, have initially shown promise in improving productivity, most have not lived up to expectations. For example, pharmacogenomics is a powerful tool for determining which patients are most likely to benefit from a given drug, but this technology has neither improved the quality of drug candidate selection nor limited candidate failure. Likewise, effective computer simulation can be a valuable tool, but is more beneficial for drug design than drug discovery. Predictive surrogates, improved biomarkers, and better animal models have proved useful for early decision making in certain situations, but, in some cases, are difficult to identify or validate and have questionable relevancy to humans.

One technology that could potentially improve the productivity and safety of drug development is Ex Vivo Metrics™. Based on ex vivo models with direct relevance to humans, Ex Vivo Metrics serves as an excellent example of the concept of research translating into practical application. Here we describe the technology, including its capabilities, advantages, and limitations.

## Ex Vivo Metrics technology

**Figure 1:**
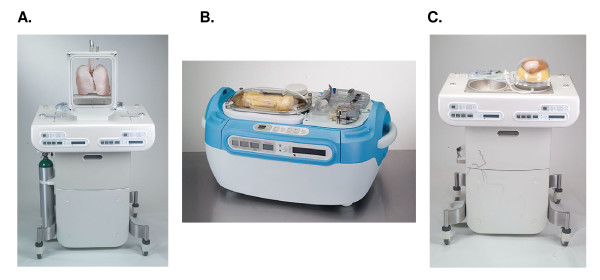
**Perfusion platforms used by Ex Vivo Metrics technology for the lungs (A), intestines (B), and liver (C)**. Each device includes an artificial thorax that meets the specific needs of the organ. For the lung (A), the artificial thorax allows for positive and negative ventilation and continuous lung function testing; for the intestines (B), it permits recording of active peristalsis; for the liver (C), it provides a heavy organ support system for reducing surface-contact pressure effects.

Ex Vivo Metrics technology is a novel, humanized preclinical test platform designed to enhance drug development. This blood-perfused system maintains isolated, intact human organs in a viable state (Figure 1), allowing compounds to be tested without the extrapolations associated with animal studies and in vitro systems or the risks associated with early human trials.

Although the development of similar perfusion systems in animals has been relatively straightforward, human whole-organ perfusion is complicated by issues related to organ procurement, transport, and revitalization. Ex Vivo Metrics uses only ethically-donated-for-research human organs made available through organ transplant programs but considered unsuitable for transplant. The donation of organs for research by individuals and families comes, rightly, with an expectation that these donated organs will be used, as far as is realistically possible, to generate relevant data that will benefit healthcare. This responsibility is not taken lightly. Organ transport is accomplished with continuous perfusion using the same techniques, equipment, and solutions employed prior to actual transplantation. For reanimation, oxygenated, matched whole blood containing the relevant biochemical substances for a given organ is perfused via the appropriate vasculature at the temperature, pressures, and flow rates that most closely replicate physiologic conditions [5]. Organs commonly and successfully perfused for Ex Vivo Metrics include the liver, intestine, and lung (Figure 1).

### The application of Ex Vivo Metrics to drug studies

For drug studies, a blood-perfused organ that meets the acceptance criteria is dosed through an appropriate route, and samples are collected for analysis (Table 1). Biopsies and blood samples are obtainable, as are tissue-specific samples, such as gut contents (intestine), bile (liver), and airway lavage (lung). The intestine and lung have been used to study drug absorption; the liver, intestine, and lung to study metabolism; and the lung to study toxicity and efficacy.

**Table 1:** Perfusion Overview

**Organs**	**Blood Input**	**Acceptance Criteria**	**Dosing Routes**	**Samples for Analysis**
**Lungs**	• Pulmonary artery • Bronchial artery	• Perfusion pressures • Flow rates • Compliance • Resistance • Airway pressures • Blood chemistry and biochemistry	• Airways • Blood	• Blood/plasma • Airway lavage • Biopsies

**Liver**	• Portal vein • Hepatic artery	• Perfusion pressures • Flow rates • Bile flow • Blood chemistry and biochemistry	• Blood	• Blood/plasma • Bile • Biopsies

**Intestine**	• Superior mesenteric artery (or its branches)	• Perfusion pressure • Flow rate • Active peristalsis • Blood chemistry and biochemistry	• Gut lumen • Blood	• Blood/plasma • Gut contents • Biopsies

To perform these studies, each organ must act as its own control. Unlike inbred animals strains matched for age, gender, diet, and environmental conditions, organs from different individuals, when exposed to drug candidates, will most certainly have quantitative differences. Often this is not appreciated until after the completion of clinical trials or during the performance of postmarketing surveillance. In practice, an organ acting as its own control means that whatever process is being studied, whether drug targeting, efficacy, and/or safety, appropriate positive and negative standards must also be applied. These standards are added before, during, or after test compounds are administered, depending on which timing is most appropriate. For example, in human gut absorption studies, passively absorbed standards with known differential rates of absorption can be coadministered with test substances for comparison. In metabolism and clearance studies, standards can be added after the majority of test substances are washed out. Likewise, for efficacy and toxicity studies, any lack of effect by new drug entities on human organs can be confirmed in the same organ by standards with well-documented activities. In this way, all blood-perfused human organs that satisfy the physiologic and biochemical criteria and are deemed viable for dosing should generate relevant, reliable, and predictable human data.

With regard to the volume of test compounds studied, it should be noted that Ex Vivo Metrics is not a high-throughput system. However, when combined with cassette dosing, this system enables thousands of drug candidates to be evaluated annually.

### Comparison to other methodologies

Ex Vivo Metrics has several advantages over other test systems currently in use for drug discovery (Table 2). The human organ perfusion model is, of course, highly relevant to humans and thus easier to extrapolate to the whole human than are whole animal and animal ex vivo perfusion studies. The Ex Vivo Metrics system is also functionally superior to tissue- and cell-based assays because it provides a full complement of intact physiologic functions and conditions, including the presence of the extracellular matrix, vasculature, and hormonal and other endogenous substances. In the future, Ex Vivo Metrics will be adapted to study diseased organs, including those with viral or bacterial infections, inflammation, tumors, or metabolic conditions such as diabetes; this capacity, once developed, will be of tremendous value, particularly when suitable animal models are limited or unavailable [5].

**Table 2:** Comparison of Ex Vivo Metrics to Other Test Systems

	**Human Ex Vivo Metrics**	**Whole Animal**	**Animal Ex Vivo Perfusion**	**Organ Baths**	**Tissue Slices**	**Cells**	**Subcellular Systems**
Relevance to target species	●	-	-	●	●	●	●
Physiologic functions	●	●	●	-	-	-	-
Nervous	○	●	○	-	-	-	-
Hormonal (relevant)	●	●	●	●	-	-	-
Vasculature	●	●	●	-	-	-	-
Full cell complement	●	●	●	●	●	-	-
Extracellular matrix	●	●	●	●	●	-	-

Key: ● Present/applicable; ○ partially present/applicable; - not present/not applicable

Ex vivo organ studies also have several advantages over human in vivo studies in terms of drug development. For example, ex vivo organ studies allow one to observe the immediate impact of a compound at the organ level and to separate out individual components of a gross effect. This provides a greater understanding of the way in which the drug interacts with selected human systems and the effects that such systems have on each other. By comparison, in a clinical trial setting, only the overall effect of a drug may be observed. Perfusion of isolated organs also permits greater control over the drug concentration applied to the organ and over other parameters related to the perfusate and the organ. For example, the contributions of diet, age, and drug dose can be systematically evaluated [5]. Multiple compounds can also be tested simultaneously to identify the potential for drug-drug interactions [5]. Advantages related to data collection include the ability to continuously monitor the system during experimentation and the ease of sample collection. First-pass or recirculated samples of effluate are straightforward to obtain, as are tissue biopsies.

Ex Vivo Metrics technology also has certain limitations. One limitation of this technology is that the perfused organs are severed from the host immune and central nervous systems. Some toxicity outcomes, such as bacterial and viral infections, involve an interplay between the invading organism and host immunity, particularly cell-mediated immunity. In situations such as these, the chosen human organs may not fully simulate natural infections. However, some aspects of the immune system and nervous system are maintained or can be simulated with Ex Vivo Metrics. For example, an appropriate innate immune response is present in some organs, and the perfusion systems allow immune cells from histocompatible individuals to be infused in a controlled manner to evaluate their contribution to toxicity. Similarly, the contribution of nerve stimulation to toxicity can be reproduced by electrical stimulation of the appropriate nerves. However, these studies have not yet been evaluated in drug candidate targeting, efficacy, and safety.

### Aims and outcomes of Ex Vivo Metrics in drug studies

Ex Vivo Metrics serves as an example of translational medicine in action through its use of basic research to potentially enhance the drug development process. An important aim of Ex Vivo Metrics is more efficient drug candidate selection, facilitated by testing compounds in human organs prior to clinical trials, so that possible failures can be eliminated at an earlier stage before significant expenditure has been made. Ex Vivo Metrics could also be applied to early-stage drug discovery to select, rank, and profile drug candidates. The technology could be used to generate metabolic, safety, and drug-drug interaction data complementary to information obtained through other systems.

Ex Vivo Metrics also offers the safety reassurances of allowing drugs to be tested and human data generated prior to human exposure and its associated risks. This is especially critical for humanized biologicals, for which traditional preclinical assessments may not be particularly informative. The ex vivo isolated blood-perfused human organ model also can help to explain divergent clinical and preclinical outcomes. For example, the system can be used to further study a drug candidate that fails in clinical trials after yielding promising results in preclinical studies to investigate the reasons why the failure occurred; this new understanding can then be applied to future candidate selections. Finally, in certain situations, Ex Vivo Metrics can function as an efficacy model when alternatives are not readily available or when knowledge of pharmacodynamic effects in a human setting is considered to be particularly important.

## Conclusion

Addressing the productivity and safety challenges of drug development will require a multidisciplinary approach, asking the right "go/no-go" questions, and having available the right tools to answer these questions in a reliable and relevant way. In addition to the tools already being used effectively, such as biomarkers, ex vivo human whole organ perfusion could potentially help to bridge the gap between preclinical and clinical drug development phases by providing specific and directly relevant pharmacokinetic, safety, and efficacy data before human trials begin. Such a system could allow rapid selection among promising drug candidates, resulting in increased productivity, and could promote increased safety. However, for Ex Vivo Metrics to be of maximum value to translational medicine, it will be necessary to prove that this technology (1) meets reliability, reproducibility, and predictability expectations; (2) provides robust and easily interpreted data; and (3) delivers tangible efficiency and effectiveness gains in a realistic time frame.

## Competing interests

Nine-TZ Healthcare Ventures is retained by Bowman Research, Inc., and receives a fee for provision of services.

## Authors' contributions

HS provided commentary on translational medicine, its challenges, and potential roles for Ex Vivo Metrics in addressing these challenges.

GC led the development and testing of Ex Vivo Metrics and provided commentary on the system.

KB provided commentary on the application of Ex Vivo Metrics to the pharmaceutical and biotechnology industries.
